# Anger under Control: Neural Correlates of Frustration as a Function of Trait Aggression

**DOI:** 10.1371/journal.pone.0078503

**Published:** 2013-10-18

**Authors:** Christina M. Pawliczek, Birgit Derntl, Thilo Kellermann, Ruben C. Gur, Frank Schneider, Ute Habel

**Affiliations:** 1 Department of Psychiatry, Psychotherapy and Psychosomatics, Medical School, RWTH Aachen University, Aachen, Germany; 2 JARA - Translational Brain Medicine, Aachen, Germany; 3 Neuropsychiatry Division, Department of Psychiatry, University of Pennsylvania and the Philadelphia Veterans Administration Medical Center, Philadelphia, Pennsylvania, United States of America; George Mason University / Krasnow Institute for Advanced Study, United States of America

## Abstract

Antisocial behavior and aggression are prominent symptoms in several psychiatric disorders including antisocial personality disorder. An established precursor to aggression is a frustrating event, which can elicit anger or exasperation, thereby prompting aggressive responses. While some studies have investigated the neural correlates of frustration and aggression, examination of their relation to trait aggression in healthy populations are rare. Based on a screening of 550 males, we formed two extreme groups, one including individuals reporting high (n=21) and one reporting low (n=18) trait aggression. Using functional magnetic resonance imaging (fMRI) at 3T, all participants were put through a frustration task comprising unsolvable anagrams of German nouns. Despite similar behavioral performance, males with high trait aggression reported higher ratings of negative affect and anger after the frustration task. Moreover, they showed relatively decreased activation in the frontal brain regions and the dorsal anterior cingulate cortex (dACC) as well as relatively less amygdala activation in response to frustration. Our findings indicate distinct frontal and limbic processing mechanisms following frustration modulated by trait aggression. In response to a frustrating event, HA individuals show some of the personality characteristics and neural processing patterns observed in abnormally aggressive populations. Highlighting the impact of aggressive traits on the behavioral and neural responses to frustration in non-psychiatric extreme groups can facilitate further characterization of neural dysfunctions underlying psychiatric disorders that involve abnormal frustration processing and aggression.

## Introduction

According to the frustration-aggression hypothesis, a feeling of frustration (thus a sense of tension, which occurs when our efforts to reach a desired goal are thwarted) evokes negative affect and anger [1,2] and therefore can lead to aggression [3–5]. Furthermore, intentionally elicited frustration (by a research assistant, for instance) has been found to increase state hostility [6] as well as state anger and affective aggression [7,8]. Anger and aggression are often closely tied in questionnaire studies of traits [9], and their underlying physiology [10] and neurochemistry [11] are merged. Therefore, in this article we will address anger and aggression and their neural correlates mostly together.

In previous behavioral and fMRI studies, frustration has been investigated using various tasks [12–15]. On a neuronal level, frustration processing was linked to (1) the dorsal anterior cingulate cortex (dACC) and the right ventral prefrontal cortex (rvPFC) during social exclusion [15,16], (2) the amygdala, the dorsolateral prefrontal cortex (dlPFC) and rostral ACC activity during defection or loss of social cooperation in a prisoner’s dilemma game [17,18] and (3) the right anterior insular cortex as well as the right inferior PFC [12], the medial PFC and ACC [19] during omission of reward. All these studies only included healthy individuals and did not provide evidence of frustration-induced negative affect.

A low tolerance for frustration and aggression are also core symptoms in several psychiatric disorders including antisocial personality disorder (ASPD) and psychopathy (PP). Investigations point to a disturbance in the interplay between the PFC (more specifically the ventromedial (vm)PFC/ orbitofrontal cortex (OFC)) and the amygdala in these disorders. This interplay is important for the processing and regulation of negative emotions, including anger and aggression [20–22]. Furthermore, aggressive individuals have shown PFC deficits (i.e. in OFC, dlPFC and ACC) [20,23–26], which may lead to reduced inhibition and thus excessive subcortical activity, predisposing them to aggressive behavior..Regarding the limbic regions, however, the findings are mixed. While on the one hand studies involving individuals with aggressive behavior have revealed hyper-reactive responses to negative emotions [20,27,28], those with additional callous-unemotional traits, on the other hand, have shown hypo-reactive responses in the amygdala and the insula [21,28,29].

Neuroimaging studies on trait aggression/ anger in healthy subjects have revealed similar results, compared to those involving pathologically aggressive groups, including decreased activation in the frontal brain regions (middle frontal cortex, dlPFC, OFC) and elevated activation in the amygdala [20,26,27,30–32]. Bettencourt and colleagues [13] concluded that ‘persons high on trait aggressiveness direct greater levels of aggressive behavior toward others even when situations are relatively neutral [which] may suggest that they have the capacity to engage in cold-blooded style of aggressive behavior’. An important advantage of using non-clinical subjects is the absence of possible confounding factors such as substance abuse, a history of child abuse or incarceration as well as comorbid psychiatric diagnoses. 

To the best of our knowledge, no study till date has examined neural processing of frustration in healthy individuals with high and low trait aggression. To this end, we used a frustration task involving solvable and unsolvable anagrams [33], which had previously revealed increased activity in the frontal and temporal regions during both conditions, with only the unsolvable anagrams increasing cerebral blood flow in the amygdala [33].

Based on previous studies, we expected (1) more frustration and thus higher reports of negative affect and anger after the task in the group with high (HA) compared to the one with low trait aggression (LA) [13], (2) increased activation in the frontal, temporal and limbic regions, including the amygdala and the insula, during processing of unsolvable anagrams in both groups [12,18,19,33] and (3) frontal regions (i.e. OFC, dlPFC, vmPFC, inferior PFC/ vlPFC and ACC) to be activated to a lesser extent in HA compared to LA [21,26,27,31,32]. (4) Amygdala activation was hypothesized to be higher in HA, thus reflecting the group's increased affective reaction to unsolvable anagrams, as reported in previous studies relating higher amygdala activation to negative events [34,35] and to more trait anger [27]. Due to the interconnection between the amygdala and the insula and their interaction during negative emotion processing [12,14,34], we also expected higher insula activation in HA. 

Finally, based on the above-mentioned findings of hypo- and hyper-reactivity in the limbic regions depending on the presence of PP traits, we aimed to further characterize our sample regarding possible concurrent PP characteristics by incorporating the Psychopathy Personality Inventory Revised [36,37]. We assumed that the group high on trait aggression would rather resemble aggressive individuals without additional primary PP traits. As a result, we expected to find higher scores on factor 2, the antisocial impulsivity scale, which relates to impulsive behavior and reactive anger in HA. No group difference was hypothesized on factor 1, the fearless dominance scale, which is more related to primary PP and the callous-unemotional trait [17,38]. 

## Methods

### 2.1: Participants

550 male students from different faculties of RWTH Aachen University completed the Aggression Questionnaire (AQ) [39]. In order to be classified as high (HA) or low (LA) in trait aggression, participants had to score above the 85^th^ or below the 15^th^ percentile respectively. Participants had no history of psychiatric or neurological disorders as assessed via a short version of the SCID I (Structured Clinical Interview for DSM-IV) [40]. The final sample consisted of 40 right-handed, native German speaking males (mean age: 22.4 (2.2)). There were 21 participants in the HA and 19 participants in the LA group. The ethics committee of the Medical Faculty of RWTH Aachen University approved the study (code EK 011/09). All individuals gave written informed consent according to the Declaration of Helsinki prior to the examination. Parts of these data have been previously reported in two short book chapters [41,42]. 

While HA and LA males differed in their reported AQ scores, for all other variables, such as age, education and various neuropsychological measures, no significant differences emerged (Table **1**). 

**Table 1:** Demographic, neuropsychology and personality data of HA and LA participants.

**Variable**	**HA (n = 21)**	**LA (n = 18)**	**t**	**df**	**p-value**
Age (years)	22.2 (2.2)	22.6 (2.2)	- .510	37	0.613
MWT-B (IQ)^a^	106.4 (11.9)	110.8 (11.4)	- 1.187	37	0.243
TMT-A^b^	22.4 (7.3)	21.4 (5.8)	.462	37	0.647
TMT-B^b^	38.4 (11.4)	38.3 (12.5)	.028	36	0.978
Verbal Fluency^c^	21.5 (6.5)	24.8 (6.0)	-1.649	37	0.108
AQ (total score) ***	86.6 (9.8)	52.4 (4.4)	14.359	28.516	0.000
Physical aggression***	26.2 (5.8)	13.8 (2.3)	8.990	26.943	0.000
Verbal aggression***	17.2 (2.8)	13.2 (2.1)	5.148	37	0.000
Anger***	20.3 (3.8)	10.7 (2.9)	8.735	37	0.000
Hostility***	22.9 (5.0)	14.7 (2.9)	6.278	33.056	0.000

Values are presented as means (s.d.). p-value Bonferroni corrected for 5 tests: 0.01.

Degrees of freedom (df) have decimals when the Levene’s test for the equality of variances is significant.

*** p<.0.001

^a^ MWT-B [79]; ^b^ Trail Making Test, form A and B [80]; ^c^ RWT [81]

### 2.2: Personality Questionnaires

To assess aggressive tendencies in more detail, participants filled in the Psychopathic Personality Inventory Revised (PPI-R) [36,37], the Life History of Aggression scale (LHA) [23] and the Freiburger Aggression Inventory (FAI) [43]. Consistent with our group division based on AQ scores and our hypotheses, HA had higher scores on the PPI-R total, PPI-R factor 2, the total scale of the FAI and three of its subscales (Table **2**). Results on the LHA revealed a marginal significant difference on total LHA score (t(37)=1.97, p=.056) with HA scoring higher compared to LA (mean LHA total score 11.43 vs. 7.94). Levels of negative affect and anger were measured with the Positive and Negative Affect Scale (PANAS) [44] and the Emotional Self Rating scale (ESR) [45] before and after the frustration task.

**Table 2:** Personality data of participants with high (HA) and low (LA) trait aggression.

**Questionnaire**	**HA (n = 21)**	**LA (n = 18)**	**t**	**df**	**p-value**
PPI-R total**	341.6 (20.8)	316.6 (25.1)	3.414	37	0.002
PPI-R_1 ^a^	113.6 (12.9)	111.9 (13.0)	.415	37	0.680
PPI-R_2 ^b**^	157.8 (24.7)	131.6 (11.8)	4.326	29.605	0.001
FAI total **	14.5 (6.6)	7.7 (4.3)	3.700	37	0.001
FAI spontaneous aggression**	4.8 (2.6)	2.0 (2.1)	3.714	36.97	0.001
FAI reactive aggression	3.8 (2.3)	2.5 (1.7)	1.911	37	0.064
FAI impulsive aggression**	5.9 (3.1)	3.2 (1.9)	3.323	33.81	0.002
FAI self-related aggression**	3.5 (3.3)	1.1 (0.9)	3.163	23.978	0.004
FAI aggression inhibition**	4.1 (2.2)	5.9 (1.6)	-2.828	37	0.008

Values are presented as means (s.d.). p-value Bonferroni corrected for 9 tests: 0.006.

Degrees of freedom (df) have decimals when the Levene’s test for the equality of variances is significant.

** p<0.01,***p<.0.001

^a^ PPI-R factor 1: fearless dominance, low behavioral inhibition; ^b^ PPI-R factor 2: antisocial impulsivity, strong behavioral activation

### 2.3: Functional frustration task

Participants were presented with 48 four-letter anagrams (24 solvable/ 24 unsolvable) of German nouns. The anagrams (white letters on black background) were shown for seven seconds. After four seconds, the participants were urged to answer by the request ‘Please respond’. Both the anagrams and the request were presented via MR compatible goggles. People responded by moving a cursor with the right hand’s index and ring fingers which were positioned on fMRI-compatible response buttons (LUMItouch™, Lightwave Technologies, Richmond, Canada). We instructed them to mark the first letter of the word they recognized by pressing a button with the right middle finger. Button press terminated the current trial. The first half of the task consisted of nineteen solvable and five unsolvable anagrams; the second half was constructed by reversing these frequencies. To further augment feelings of frustration while having to deal with the unsolvable anagrams, participants were informed that good performance would be rewarded with extra money. After each anagram, participants received feedback on their performance through the display of either a positive symbol (a smiley) and the sentence ‘You have won thirty Cents’ or a negative symbol (a frowney) and the sentence ‘You have lost thirty Cents’. The task was presented in a block design, where each block lasted 36 seconds and contained three anagrams (each anagram 7s + interstimulus interval minimum 3s + feedback 2s). After each block, a baseline (fixation cross) followed for 15s. Total duration of the task was 13.6min. The paradigm was programmed using the Presentation software package (Neurobehavioral Systems Inc., Albany, CA, USA). In order to assess possible behavioral performance differences between the groups, the number of anagrams solved and reaction times of solvable and unsolvable anagrams were measured. 

### 2.4: Behavioral data analysis

Statistical analyses were performed using SPSS 18.0 (SPSS, Inc., IL, USA) and level of significance was set at p=0.05. Group differences on demographic, personality and behavioral data (accuracy and reaction times on solvable and unsolvable anagrams) were analyzed using independent samples t-tests. In cases of significant Levene’s test for homogeneity of variance, degrees of freedom were adapted using Satterthwaite’s correction. Results are corrected for multiple testing using Bonferroni correction. Repeated measures ANOVAs were applied with negative affect (PANAS pre and PANAS post) and anger (ESR pre and ESR post) as within-subjects factor and trait aggression (HA vs. LA) as between-subjects factor. For significant differences, estimates of effect size are given as partial η^2^ and Cohen’s d.

### 2.5: Functional MRI acquisition parameters

Functional scanning was performed on a Siemens Trio 3 Tesla magnetic resonance scanner. For the blood oxygen level dependent (BOLD)-sensitive MRI measurement, we used a T2*-weighted gradient echo sequence with the following parameters: TR = 2500, TE = 30ms, FoV = 200 mm, 38 axial slices (whole brain coverage), slice thickness = 3.1 mm, in-plane-resolution = 3.125 x 3.125 x 3.1 mm, flip-angle = 77°, Matrix size 64 x 64, slice gap = 0.31 mm. A total of 350 functional images parallel to the intercommissural line (anterior-posterior commissure) with an interleaved order of slice acquisition were acquired on each participant. Four dummy scans were acquired to allow steady-state magnetization and were discarded from further analysis After functional neuroimaging, a 4 min. magnetization-prepared rapid acquisition gradient echo image (MP-RAGE) T1-weighted sequence was applied to obtain structural images (TR = 1900 ms, TE = 2.52 ms, TI = 900 ms, matrix = 256 x 256, 176 slices, FoV: 250 x 250 mm^2^, flip angle = 9°, voxel size = 1 x 1 x 1 mm^3^). 

### 2.6: Functional MRI data analysis

Functional data were preprocessed and analyzed using SPM5 [46] (http://www.fil.ion.ucl.ac.uk/spm/spm5.html). Images of each subject were realigned to the mean image (after a first-pass realignment on the first image of the time-series) to correct for head motion, normalized into the standardized stereotactic space (interpolation to a resolution of 2 x 2 x 2mm^3^) and the functional data sets were spatially smoothed using an isotropic Gaussian kernel with a full-width-at-half-maximum of 8 mm^3^. 

At the first level, a separate GLM was specified for each participant. The model included separate regressors for solvable (8 blocks) and unsolvable (8 blocks) anagrams, which were convolved with the canonical hemodynamic response function. Further, we entered the six realignment parameters as covariates of no interest in the first-level analysis. Data were high-pass filtered with a cut-off of 128 s to remove low-frequency drifts. Serial correlations were accounted for by a first-order autoregressive model. 

In order to assess differences between the two extreme groups, contrast images of solvable and unsolvable conditions from all participants were included in a second-level random-effects analysis. Activation differences in brain regions were examined by a mixed effects two-way ANOVA with group (HA vs. LA) as between-subjects factor and condition (solvable vs. unsolvable anagrams) as within-subjects factor in order to detect significant main or interaction effects. The resulting statistical maps for the main effects of group and condition and the group x condition interaction (all F-contrasts) effect were thresholded at p<0.001 uncorrected with a voxel extent of 20 contiguous voxels (for illustration purposes) as has been applied in previous studies [12,47,48]. Stereotaxic coordinates of local maxima of activation are expressed as x;y;z values in proper MNI space. Anatomical localizations were identified using the Anatomy Toolbox [49,50] and the WFU Pick Atlas as tools implemented in SPM. Concerning the division of the frontal lobe, we closely correspond to [26].

### 2.7: Region of Interest (ROI) analysis

We performed an ROI analysis for the left and right amygdala with to maximize sensitivity to group differences in this region. We specifically aimed at investigating the amygdala’s role during frustration because of its function in emotion processing, trait anger [27] and in similar task [33]. Values for amygdala ROIs were extracted based on probabilistic cytoarchitectonic maps [51], as available in the Anatomy Toolbox in SPM5 [49,50]. Mean parameter estimates were extracted for left and right amygdala in both conditions (solvable and unsolvable). Levene’s test for homogeneity of variances indicated homoscedasticity for all parameter estimates (solvable left: p=0.167; solvable right: p=0.058; unsolvable left: p=0.322; unsolvable right: p=0.389). Three-way ANOVAs were applied for the left and right amygdala with group as between-subjects factor and condition and laterality as within-subjects factors. 

### 2.8: Corollary analyses

Correlation analyses were performed for personality measures and amygdala activation scores (mean parameter estimates taken from the ROI analysis) separately in HA and LA. Personality measures included scores on the PPI-R, FAI and the LHA. Results are regarded significant at p<0.05.

## Results

### 3.1: Behavioral and current mood data

One subject was excluded due to excessive head movement during scanning, leaving 21 HA individuals and 18 LA individuals for final analysis.

HA and LA showed no differences on number of anagrams solved (t(37)=0.046, p=0.963, two-tailed) and reaction times on solvable (t(37)=0.653, p=0.518, two-tailed) and unsolvable anagrams (t(25.702)=0.670, p=0.509, two-tailed). 

Based on the difference scores on the PANAS (post- minus pre-scores), 13 participants (62%) were classified as responders (i.e. they showed an increase in negative affect or anger levels after the frustration task) in HA, while there were only 4 such responders (22%) in LA. Analysis of PANAS data revealed marginal significant pre vs. post main effect (F(1,37)=3.638, p=0.064) as well as a marginal significant time x group interaction effect (F(1,37)=3.638, p=0.064) and a significant main effect of group (F(1,37)=8.093, p=0.007, partial η^2^=0.18), demonstrating higher levels of negative affect in HA compared to LA. Assuming a greater increase in negative affect (from pre to post) in the HA group, the independent samples t-test on the PANAS difference scores decomposed the interaction effect and was significant (t(29.099)=2.007, p=0.027, d=0.63, one-tailed), thereby revealing a greater increase of negative affect in the HA compared to the LA group. 

For the ESR data, we observed a significant pre vs. post effect (F(1,37)=5.374, p=0.026, partial η^2^=0.13) with higher values after compared to before the measurement, and group (F(1,37)=8.197, p=0.007, partial η^2^=0.18), indicating higher levels of anger in HA compared to LA. The interaction was not significant (F(1,37)=2.077, p=0.158). 

**Figure 1:**
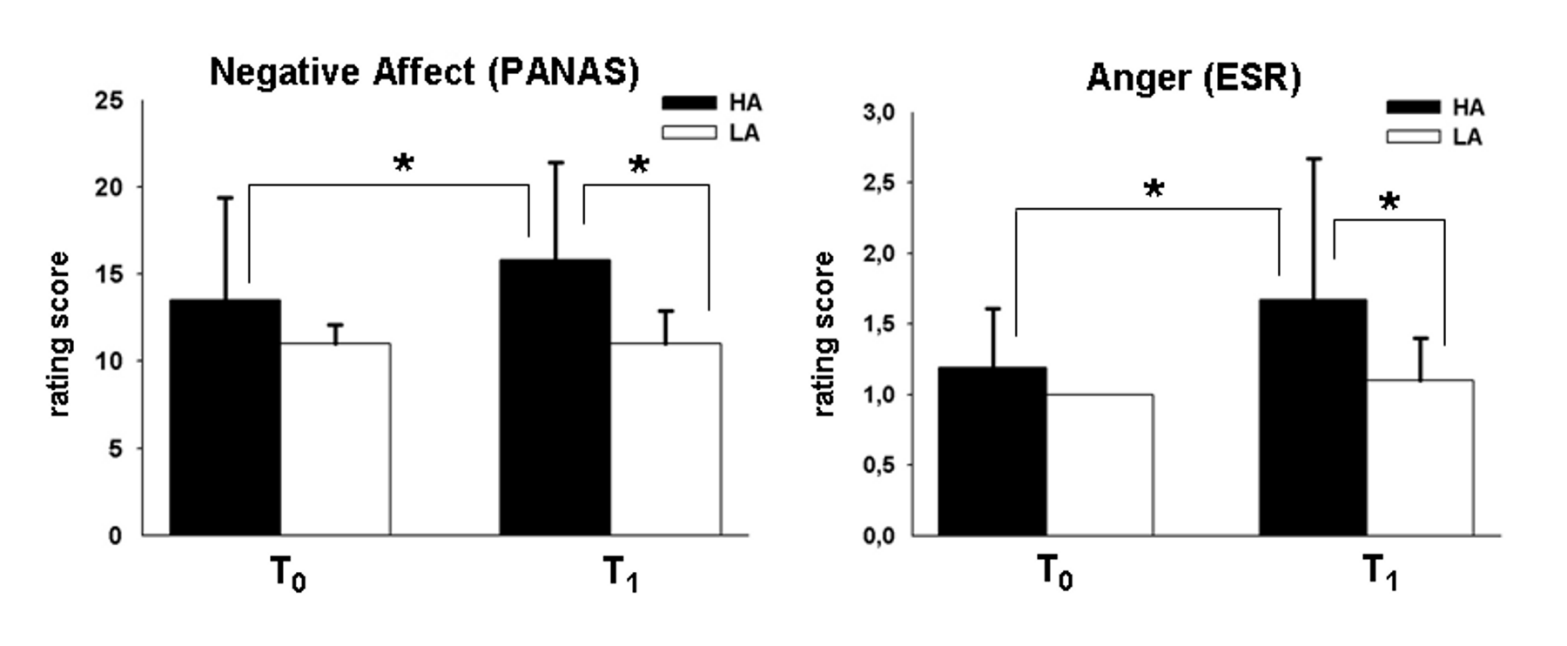
Reported level of Negative Affect (PANAS) and Anger (ESR) by individuals with high trait aggression (HA) and low trait aggression (LA) before (T0) and after (T1) the anagram task; * significant at p<0.05.

Post-hoc analyses revealed that HA individuals showed significantly increased negative affect (PANAS, t(20)=2.24, p=0.036, two-tailed) and anger ratings (ESR, t(20)=2.12, p=0.047, two-tailed) after the frustration task (vs. before the task), while the results from these paired samples t-tests were not significant in LA (PANAS, t(17)=.000, p=1.00, two-tailed; ESR, t(17)=1.46, p=.163, two-tailed). Direct comparison between the two groups via independent samples t-tests revealed that HA exhibited a significantly higher level of negative affect (t(25.50)=3.62, p=0.001, d=1.13, two-tailed) and anger ratings (t(25.07)=2.48, p=0.020, d=0.78, two-tailed) compared to LA after the task (Figure ***1***)*.*

Concerning the other emotions on the ESR, no significant group differences were observed (all p>0.186), except for sadness where HA revealed a trend effect before the task (t(20)=2.02, p =0.056) and significantly higher scores after the anagram task compared to LA (t(20)=2.34, p=0.030, d=0.73).

### 3.2: Functional MRI data

**Figure 2:**
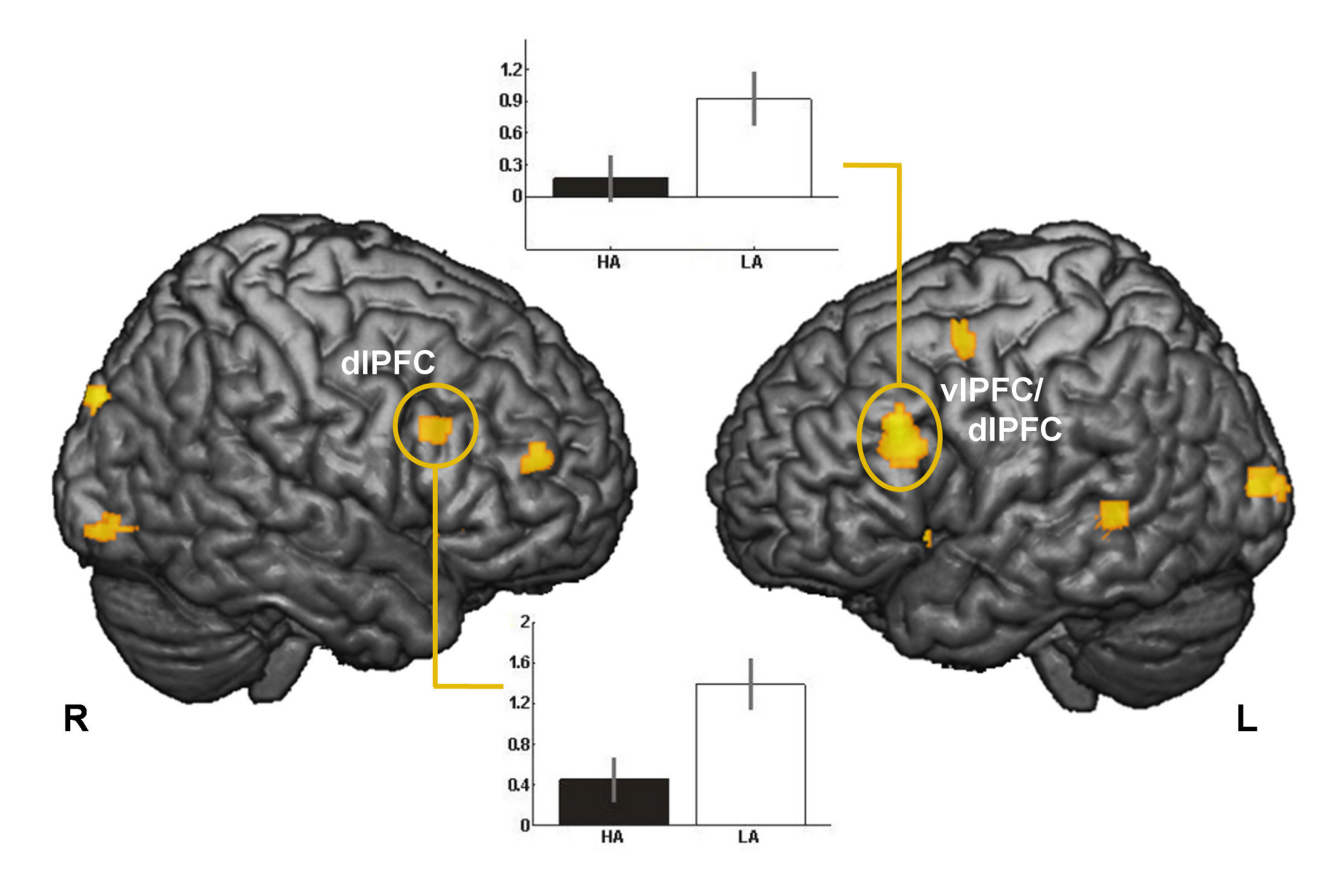
Activation for the main effect of group and parameter estimates for the activation in right dorsolateral prefrontal cortex (dlPFC, x=44, y=52, z=18) and left ventrolateral/ dorsolateral prefrontal cortex (vlPFC/ dlPFC, x=-52, y=26, z=32), p<0.001, uncorr., k>20 voxel.

**Figure 3:**
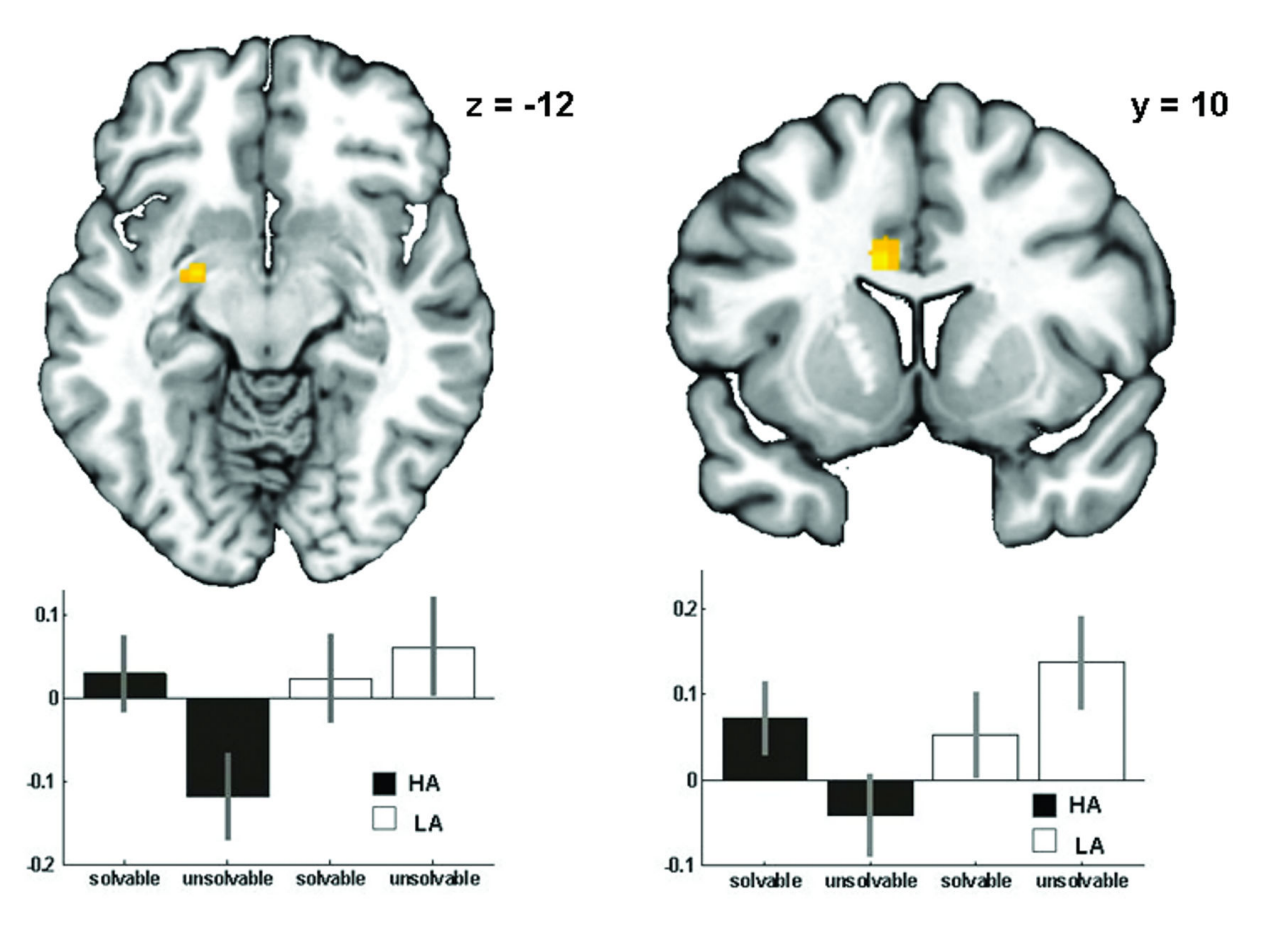
Group X condition interaction and parameter estimates for activation in the amygdala and the dACC of HA and LA. dACC: F(1,74), x=-8, y=10, z=26; amygdala (laterobasal group): F(1,74), x=-22, y=-6, z=-12; threshold: p<0.001 uncorr., k>20 voxel.

The ***maineffectofcondition*** (solvable vs. unsolvable anagrams) revealed activation in the cingulate cortex, the bilateral superior and left middle frontal cortex, the bilateral angular gyrus and the superior parietal regions (for more details see Table **S1**). The ***maineffectofgroup*** depicted activation in left vlPFC/ dlPFC, right dlPFC, right middle cingulate, right insula and left middle temporal regions (Figure **2** and detailed information in the Table **S2**). The **group X condition interaction** indicated activation differences in the left amygdala and left dACC, the right vlPFC cortex, the left parietal cortex and the right parahippocampal gyrus (Figure **3** and Table **3**). 

Analysis of parameter estimate values from the amygdala (x=-22, y=-6, z=-12), the dACC (x=-8, y=10, z=26) and the vlPFC (x=34, y=36, z=10) revealed relatively less activation in HA compared to LA during the unsolvable condition (amygdala: (t(37)=3.081, p=0.004, d=-6.13; dACC: t(37)=3.675, p=0.001, d=-1.18; vlPFC: t(37)=2.520, p=0.016, d=-0.80) but no difference between groups during the solvable condition (amygdala: (t(37)=1.080, p=0.287; dACC: t(37)=0.308, p=0.759; vlPFC: t(37)=0.640, p=0.526). Parameter estimate values for all other regions of the interaction effect can be found in Figure **S1.**

**Table 3:** Activation cluster for the group X condition interaction, p<.001 uncorr., k=20.

	**MNI Coordinates**						
**Brain region**	**x**	**y**	**z**	**side**	**k**	**Z-score**	**p-value**
dorsal anterior cingulate cortex	-8	10	26	L	56	4.54	0.000
cerebellum	0	-68	-24		41	3.97	0.000
thalamus	4	-6	0	R	38	4.07	0.000
amygdala	-22	-6	-12	L	36	4.00	0.000
inferior parietal	-34	-44	28	L	29	4.06	0.000
lateral globus pallidus	-24	-18	-2	L	29	3.80	0.000
parahippocampal gyrus	10	-6	-18	R	27	3.55	0.000
vlPFC	34	36	10	R	26	3.43	0.000
Claustrum	-26	-12	22	L	24	3.50	0.000
cingulate gyrus	-12	-10	32	L	23	3.76	0.000

Abbreviations: k = cluster size

### 3.3: ROI analysis

The repeated-measures ANOVA revealed a significant effect of condition, F(1,37)=6.513, p=0.015, η^2^=0.15, indicating stronger amygdala activation during the solvable condition. Further, a significant main effect of laterality emerged, F(1,37)=8.27, p=0.007, η^2^=0.18, pointing to higher activation levels in the left compared to the right amygdala. This is in line with earlier propositions of left-lateralization of amygdala activity during affect processing [52]. No significant main effect of group (F(1,37)=0.470, p=0.497) or interaction effect (all p>0.199) emerged. 

### 3.4: Corollary analyses

Exploratory correlation analyses revealed several moderate associations: in HA, during unsolvable anagrams, negative correlations emerged between the left amygdala and levels of reactive aggression (r=-0.575, p=0.006), FAI summary score (r=-0.442, p=0.045) as well as LHA scores (r=-0.397, p=0.075). Furthermore, levels of spontaneous aggression were negatively correlated to left amygdala activation during unsolvable anagrams (r=-0.490, p=0.024), while there was a trend for the right amygdala (r=-0.424, p=0.056). All other tested correlations failed to reach significance (all p>0.101). Specifically in LA, no correlations between amygdala levels and questionnaire data were observed. 

## Discussion

Our study was the first to look into the neural correlates of frustration and associated anger in healthy males with high and low trait aggression. Consistent with our hypothesis, HA reported significantly higher levels of negative affect and anger after the frustration task. The finding of stronger activation in LA compared to HA in the left vlPFC/ dlPFC and right dlPFC is in accordance with previous results relating frontal brain functioning to the regulation of aggression [14] and the left dlPFC to higher cognitive and self-regulatory processes, suggesting that dysfunction in this region is related to impulsivity and poor behavioral control [26]. In addition, more reactive aggression has been observed in healthy participants after theta burst magnetic stimulation over the left dlPFC [53]. Hence, a distinct frontal brain dysfunction seems to characterize both pathologically aggressive individuals and persons with high trait aggression. We speculate that this neural abnormality might have the potential to act as an endophenotype [54], which certainly deserves further study.

### Group X condition interaction effect - activation in vlPFC and dACC

As hypothesized, the interaction revealed lower activation in the left dorsal anterior cingulate cortex (dACC) and the right vlPFC in HA compared to LA while working on the unsolvable anagrams. This is in line with previous results relating lower vlPFC activation to impulsivity [55] and aggression [56]. Activation in the dorsal part of the ACC has frequently been reported in cognitive control and conflict monitoring [57,58] as well as in studies involving a conflict between cognitive and emotional motivations [14,59]. Sterzer and colleagues (2005) [48] explained the observed dACC deactivation during negative affect as interference of emotional states with cognitive processing, resulting in a failure to cognitively control and regulate emotional behavior. In line with these findings, the relatively lower activation of dACC in HA might reflect an impaired capability to control and regulate their frustration, thereby leading to a heightened propensity for impulsive aggression. While this propensity is often manifested openly in patient groups, HA subjects may have alternative strategies for counteracting the attenuated activity and the disposition to aggressive behavior. This speculation appears plausible in light of higher, albeit marginally significant, scores on total LHA in HA subjects. Total LHA has been reported to be a reliable and valid measure of a history of overt aggression in control subjects [23]. 

Though our findings are in line with some previous studies reporting ACC but not OFC activation in response to stress and frustration [12,16,19,60], the lack of OFC/vmPFC activation in our study warrants discussion. Notably, OFC/vmPFC activation was observed in studies directly inducing anger or by passively viewing angry faces [61–63] while studies eliciting frustration by omission of reward [12] or social exclusion [15] reported pronounced ACC activation. Possibly, our task primarily induced frustration rather than anger, thus explaining why we did not observe significant activation in the OFC. Furthermore, dysfunction or damage in the OFC/vmOFC has been related to deficits in emotional/ social conduct [64–66] and aggression [26,67,68] and might thus imply a clear predisposition to pathological aggressive/ antisocial behavior. In contrast, in HA subjects we found distinct activation in the regulatory areas related to impulsivity (i.e. vlPFC and dlPFC). Also, while none of the adult trait anger/ aggression studies found activation in OFC/vmPFC [27,31], studies on trait PP revealed reduced activation in the high PP group [17,47,69]. Therefore, abnormal activation in this region seems to be associated with PP traits rather than aggressive traits – at least in a healthy population.

Regarding activation in the frontal lobe, Potegal (2012) [9] refers to an important paradox. According to lesion studies characterizing the inhibition of behavior such as aggression as a general function of the frontal lobe, increased anger should lead to reduced frontal lobe activity. Nevertheless, for the most part, activation increases are reported, which might be explained by GABA interneurons inhibiting activation of the OFC, thereby leading to reduced activity in OFC output neurons, and mesolimbic dopamine feedback loops simultaneously modulating the GABA interneurons, which subsequently balance out the reduced OFC activity leading to increased activation [9].

### Interaction effect - amygdala activation

The observation of lower amygdala activation in HA with higher levels of anger was unexpected. Together with the lower frontal (i.e. dACC and vlPFC) activation in HA, this finding might reflect distinct processing strategies of the negative mood state in HA subjects. The prefrontal cortex has interconnections with the amygdala and thereby can modulate its activity [21,34,70,71]. This mechanism has been observed in disorders marked by impulsive aggression [22,72] and violence [20]. Since the general working model is that of prefrontal activation inhibiting the amygdala, e.g. [71], the attenuated activation in both the frontal cortex and the amygdala might seem surprising. A possible explanation could be an uncoupling or dysfunctional connectivity between the amygdala and the frontal cortex, or, alternatively, the involvement of a modulator region (e.g. the rostral ACC) inhibiting both the amygdala and the frontal cortex in HA. These possibilities should be explored through connectivity studies in larger clinical groups or individuals with extreme personality traits during emotion regulation tasks. 

Further, attenuated amygdala response has been reported in a range of aggressive populations, especially PP and PP traits [17,20,67,69,73,74]. Lower amygdala activation in HA has been found to be associated with higher levels of spontaneous and reactive aggression. In this regard, Osumi and colleagues have recently reported higher psychopathic tendencies in healthy males being related to attenuated amygdala activity during a task triggering reactive aggression [74]. Still, we need to be cautious here as our groups differed only on additional aggression measures (FAI, PPI-R total score and PPI-R factor 2). There was no difference on PPI-R factor 1, which is related to primary PP and the callous-unemotional trait [17,38]. Aggressive individuals without additional primary PP traits have shown hyper-reactive responses in negative emotion processing [15,22,25], which is contrary to our findings. Alternatively, it could be speculated that the lower amygdala activation does not only reflect self-reported levels of anger in response to frustration in HA subjects, but also a reduced capability of impulse inhibition and lack of affective controllability. However, the observation involving the amygdala is hard to interpret and the possibility remains that there are differential underlying mechanisms in HA and LA. While the HA subjects felt frustrated during the unsolvable anagrams, the LA group might have experienced a pleasant challenge.

In sum, our results resemble previous findings in abnormally aggressive individuals, emphasizing the role of the frontal cortex, the dACC, the amygdala and partly the insula in frustration processing and the resulting feelings of anger. The insula was found to be less activated in HA, which however could not be specifically linked to the processing of unsolvable anagrams. Based on our findings, which suggest distinct frontal and limbic processing mechanisms of frustration as a function of trait aggression, further research on aggression as a dimensional construct can lead to better understanding and consequently help reduce or prevent aggression and violence in clinical populations.

### Limitations and future investigations

While this study provides new insight into the neural correlates of frustration and the impact of trait aggression, several methodological constraints have to be considered: The use of a non-frustration task that elicits another emotion (e.g. fear) would have been helpful in further disentangling the brain responses that are specific to frustration from those that are generally linked to emotion reactivity and regulation.

Further, a study stated that the groups in an extreme group approach (EGA) [75] should contain 1/4 to 1/3 of the data [76]. However, we had to consider the cost-time-efficiency in our study since fMRI studies are both costly and time consuming. Also, we used EGA to detect hypothesized effects between groups which were drawn from a healthy population and therefore the expected effects were quite small. Our results, therefore, are limited compared to an analysis of full-range, continuous data [75]. The range for correlation analyses was also restricted as we performed these analyses within groups, due to the EGA.

While the use of non-pathological extreme groups has its benefits (e.g. the absence of substance abuse, a history of child abuse), there might be important attributes of a pathological group regarding behavior, reaction and neural processing of anger and frustration that could not be explored or taken advantage of in our study. Overt aggression possibly constitutes the factor that particularly differentiates between healthy individuals with high trait aggression and abnormally aggressive individuals. Therefore, future studies incorporating a third group showing a pathological level of aggression can compare the groups on their underlying processing mechanisms of anger and frustration and related behavioral and personality characteristics. 

Finally, earlier studies [77], for an overview [78] have also implemented autonomic measures, which afford further insight into the psychophysiological processes underlying frustration and anger. Separate and simultaneous measurement of felt anger, anger control effort, facial expressions, as well as aggressive impulses and action also warrants future investigation. All these studies aim at determining the underlying mechanisms of anger and aggression and gathering information on the pathophysiology of psychiatric disorders involving aggression and abnormal frustration processing.

## Supporting Information

Table S1 **Activations for the main effect of condition, p** **<**.001** uncorr., k=20.**
(DOCX)

Table S2 **Activations for the main effect of group, p** **<**.001** uncorr., k=20.**
(DOCX)

Figure S1 **Parameter estimate plots for the activation cluster of the group X condition interaction.**
(DOCX)

## References

[1] Berkowitz RL, Harmon-Jones E (1989) Frustration–aggression hypothesis: Examination and reformulation. Psychol Bull 106: 59–73. doi:10.1037/0033-2909.106.1.59. PubMed: 2667009. 2667009

[2] Berkowitz RL, Harmon-Jones E (1990) On the formation and regulation of anger and aggression: A cognitive–neoassociationistic analysis. Am Psychol 45: 494–503. doi:10.1037/0003-066X.45.4.494. PubMed: 2186678. 2186678

[3] Nelson RJ, Trainor BC (2007) Neural mechanisms of aggression. Nat Rev Neurosci 8: 536-546. doi:10.1038/nrn2174. PubMed: 17585306. 17585306

[4] Patrick CJ, Cuthbert BN, Lang PJ (1994) Emotion in the criminal psychopath: fear image processing. J Abn Psychol 103: 523–534. doi:10.1037/0021-843X.103.3.523. PubMed: 7930052. 7930052

[5] Siever LJ (2008) Neurobiology of aggression and violence. Am J Psychiatry 165: 429-442. doi:10.1176/appi.ajp.2008.07111774. PubMed: 18346997. 18346997 PMC4176893

[6] Williams KD (2009) The Effects of frustration, violence, and trait hostility after playing a violent video game. Mass Commun Soc 12: 291-310. doi:10.1080/15205430802461087.

[7] Berkowitz RL, Harmon-Jones E (2004) Toward an understanding of the determinants of anger. Emotion 4: 107-130. doi:10.1037/1528-3542.4.2.107. PubMed: 15222847. 15222847

[8] Harmon-Jones E, Sigelman J (2001) State anger and prefrontal brain activity: Evidence that insult-related relative left-prefrontal activation is associated with experienced anger and aggression. J Pers Soc Psychol 80: 797-803. doi:10.1037/0022-3514.80.5.797. PubMed: 11374750. 11374750

[9] Potegal M (2012) Temporal and frontal lobe initiation and regulation of the top-down escalation of anger and aggression. Behav Brain Res 231: 386-395. doi:10.1016/j.bbr.2011.10.049. PubMed: 22085875. 22085875

[10] Williams JE (2010) Anger/hostility and cardiovascular disease. In: Potegal M International handbook of anger. New York: Springer Verlag pp. 435-447.

[11] Bond A, Wingrove J (2010) The neurochemistry and psychopharmacology of agner. In: Potegal M International handbook of anger. New York: Springer Verlag pp. 79-102.

[12] Abler B, Walter H, Erk S (2005) Neural correlates of frustration. Neuroreport 16: 669-672. doi:10.1097/00001756-200505120-00003. PubMed: 15858403. 15858403

[13] Bettencourt BA, Talley A, Benjamin AJ, Valentine J (2006) Personality and aggressive behaviour under provoking and neutral conditions: A meta-analytic review. Psychol Bull 132: 751-777. doi:10.1037/0033-2909.132.5.751. PubMed: 16910753. 16910753

[14] Denson TF, Pedersen WC, Ronquillo J, Nandy AS (2008) The angry brain: Neural correlates of anger, angry rumination, and aggressive personality. J Cogn Neurosci 21: 734-744. PubMed: 18578600. 10.1162/jocn.2009.21051 18578600

[15] Eisenberger NL, Lieberman MD, Williams KD (2003) Does rejection hurt? An fMRI study of social exclusion. Science 302: 290-292. doi:10.1126/science.1089134. PubMed: 14551436. 14551436

[16] Eisenberger NI, Taylor SE, Gable SL, Hilmert CJ, Lieberman MD (2007) Neural pathways link social support to attenuated neuroendocrine stress responses. NeuroImage 35: 1601-1612. doi:10.1016/j.neuroimage.2007.01.038. PubMed: 17395493. 17395493 PMC2710966

[17] Rilling JK, Glenn AL, Jairam MR, Pagnoni G, Goldsmith DR et al. (2007) Neural correlates of social cooperation and non-cooperation as a function of psychopathy. Biol Psychiatry 61: 1260-1271. doi:10.1016/j.biopsych.2006.07.021. PubMed: 17046722. 17046722

[18] Rilling JK, Gutman DA, Zeh TR, Pagnoni G, Berns GS et al. (2002) A neural basis for social cooperation. Neuron 35: 395-405. doi:10.1016/S0896-6273(02)00755-9. PubMed: 12160756. 12160756

[19] Siegrist J, Menrath I, Stöcker T, Klein M, Kellermann T et al. (2005) Differential brain activation according to chronic social reward frustration. Neuroreport 16: 1899-1903. doi:10.1097/01.wnr.0000186601.50996.f7. PubMed: 16272875. 16272875

[20] Coccaro EF, McCloskey MS, Fitzgerald DA, Phan KL (2007) Amygdala and orbitofrontal reactivity to social threat in individuals with impulsive aggression. Biol Psychiatry 62: 168-178. doi:10.1016/j.biopsych.2006.08.024. PubMed: 17210136. 17210136

[21] Coccaro EF, Sripada CS, Yanowitch RN, Phan KL (2011) Corticolimbic function in impulsive aggressive behavior. Biol Psychiatry 69: 1153-1159. doi:10.1016/j.biopsych.2011.02.032. PubMed: 21531387. 21531387

[22] New AS, Hazlett EA, Newmark RE, Zhang J, Triebwasser J et al. (2009) Laboratory Induced Aggression: A Positron Emission Tomography Study of Aggressive Individuals with Borderline Personality Disorder. Biol Psychiatry 66: 1107-1114. doi:10.1016/j.biopsych.2009.07.015. PubMed: 19748078. 19748078 PMC2788117

[23] Coccaro EF, Berman ME, Kavoussi RJ (1997) Assessment of life history of aggression: development and psychometric characteristics. Psychiatry Res 73: 147-157. doi:10.1016/S0165-1781(97)00119-4. PubMed: 9481806. 9481806

[24] Raine A, Buchsbaum MS, Stanley J, Lottenberg S, Abel L et al. (1994) Selective reductions in prefrontal glucose metabolism in murderers. Biol Psychiatry 36: 365-373. doi:10.1016/0006-3223(94)91211-4. PubMed: 7803597. 7803597

[25] Raine A, Meloy JR, Bihrle S, Stoddard J, LaCasse L et al. (1998) Reduced prefrontal and increased subcortical brain functioning assessed using positron emission tomography in predatory and affective murderers. Behav Sci Law 16: 319-322. doi:10.1002/(SICI)1099-0798(199822)16:3. PubMed: 9768464. 9768464

[26] Yang Y, Raine A (2009) Prefrontal structural and functional brain imaging findings in antisocial, violent and psychopathic indiviuals: a meta-analysis. Psychiatry Res 174: 81-88. doi:10.1016/j.pscychresns.2009.03.012. PubMed: 19833485. 19833485 PMC2784035

[27] Carre JM, Fisher PM, Manuck SB, Hariri AR (2010) Interaction between trait anxiety and trait anger predict amygdala reactivity to angry facial expressions in men but not women. Soc Cogn Affec Neurosci 2: 213-221. 10.1093/scan/nsq101 PMC3277369 21183456

[28] Sebastian CL, McCrory EJP, Cecil CAM, Lockwood PL, De Brito SA et al. (2012) Neural responses to affective and cognitive theory of mind in children with conduct problems and varying levels of callous-unemotional traits. Arch Gen Psychiatry 69: 814-822. doi:10.1001/archgenpsychiatry.2011.2070. PubMed: 22868935. 22868935

[29] Jones AP, Laurens KR, Herba CM, Barker GJ, Viding E (2009) Amygdala hypoactivity to fearful faces in boys with conduct problems and callous-unemotional traits. Am J Psychiatry 166: 95-102. doi:10.1176/appi.ajp.2008.07071050. PubMed: 18923070. 18923070

[30] Birbaumer N, Veit R, Lotze M, Erb M, Hermann C et al. (2005) Deficient fear conditioning in psychopathy. Arch Gen Psychiatry 62: 799-805. doi:10.1001/archpsyc.62.7.799. PubMed: 15997022. 15997022

[31] Pawliczek CM, Derntl B, Kellermann T, Kohn N, Gur RC et al. (2013) Inhibitory control and trait aggression: neural and behavioral insights using the emotional stop signal task. NeuroImage 79: 264-274. doi:10.1016/j.neuroimage.2013.04.104. PubMed: 23660028. 23660028

[32] Strenziok M, Krueger F, Heinecke A, Lenroot RK, Knutson KM et al. (2011) Developmental effects of aggressive behavior in male adolescents assessed with structural and functional brain imaging. Soc Cogn Affec Neurosci 6: 2-11. doi:10.1093/scan/nsp036. PubMed: 19770220. PMC3023076 19770220

[33] Schneider F, Gur RE, Alavi A, Seligman ME, Mozley LH et al. (1996) Cerebral blood flow changes in limbic regions induced by unsolvable anagram tasks. Am J Psychiatry 153: 206-212. PubMed: 8561200. 8561200 10.1176/ajp.153.2.206

[34] Carretié L, Albert J, López-Martín S, Tapia M (2009) Negative brain: An integrative review on the neural processes activated by unpleasant stimuli. Int J Psychophysiol 71: 57-63. doi:10.1016/j.ijpsycho.2008.07.006. PubMed: 18727941. 18727941

[35] Costafreda SG, Brammer MJ, David AS, Fu C (2008) Predictors of amygdala activation during the processing of emotional stimuli: A meta-analysis of 385 PET and fMRI studies. Brain. Res Rev 58: 57-70. doi:10.1016/j.brainresrev.2007.10.012. 18076995

[36] Alpers GW, Eisenbarth H, editors (2008) Psychopathic Personality Inventory-Revised (PPI-R). Deutsche Version. Goettingen.

[37] Lilienfeld SO, Andrews BP (1996) Development and preliminary validation of a self-report measure of psychopathic personality traits in noncriminal populations. J Pers Assess 66: 488-524. doi:10.1207/s15327752jpa6603_3. PubMed: 8667144. 8667144

[38] Benning SD, Patrick CJ, Blonigen DM, Hicks BM, Iacono WG (2005) Estimating facets of psychopathy from normal personality traits: A step toward community-epidemiological investigations. Assessment 12: 3-18. doi:10.1177/1073191104271223. PubMed: 15695739. 15695739 PMC2242356

[39] Buss AH, Perry M (1992) The aggression questionnaire. J Pers Soc Psychol 63: 452-459. doi:10.1037/0022-3514.63.3.452. PubMed: 1403624. 1403624

[40] Wittchen HU, Zaudig M, Fydrich T, editors (1997) Strukturiertes Klinisches Interview für DSM-IV. Goettingen: Hogrefe.

[41] Pawliczek C (2012) Neuronale Korrelate von Frustration und Impulsivität. In: Schneider F Positionen der Psychiatrie. Heidelberg: Springer Verlag.

[42] Pawliczek C, Habel U (2012) Neuronale Korrelate von Frustration bei Maennern mit hoher und niedriger Trait-Aggressivität; In: Müller JL Roesler M Briken P Fromberger P Jordan K EFPPP Jahrbuch 2012 Empirische Forschung in der forensischen Psychiatrie, Psychologie und Psychotherapie. Goettingen: Medizinisch Wissenschaftliche Verlagsgesellschaft

[43] Hampel R, Selg H (1975) Der Freiburger Aggressionsfragebogen (FAF). Göttingen: Hogrefe.

[44] Krohne HW, Egloff B, Kohlmann C-W, Tausch A (1996) Untersuchung mit einer deutschen Form der Positive and Negative Affect Schedule (PANAS). Diagnostica 42: 139-156.

[45] Schneider F, Gur RC, Gur RE, Muenz LR (1994) Standardized mood induction with happy and sad facial expressions. Psychiatry Res 51: 19-31. doi:10.1016/0165-1781(94)90044-2. PubMed: 8197269. 8197269

[46] Friston KJ, Holmes AP, Worsley KJ, Poline J-P, Frith CD et al. (1994) Statistical parametric maps in functional imaging: A general linear approach. Hum Brain Mapp 2: 189–210. doi:10.1002/hbm.460020402.

[47] Fullam RS, McKie S, Dolan MC (2009) Psychopathic traits and deception: functional magnetic resonance imaging study. Br J Psychiatry 194: 229-235. doi:10.1192/bjp.bp.108.053199. PubMed: 19252152. 19252152

[48] Sterzer P, Stadler C, Krebs A, Kleinschmidt A, Poustka F (2005) Abnormal neural responses to emotional visual stimuli in adolescents with conduct disorder. Biol Psychiatry 57: 7-15. doi:10.1016/j.biopsych.2004.10.008. PubMed: 15607294. 15607294

[49] Eickhoff SB, Heim S, Zilles K, Amunts K (2006) Testing anatomically specified hypotheses in functional imaging using cytoarchitectonic maps. NeuroImage 32: 570-582. doi:10.1016/j.neuroimage.2006.04.204. PubMed: 16781166. 16781166

[50] Eickhoff SB, Stephan KE, Mohlberg H, Grefkes C, Fink GR et al. (2005) A new SPM toolbox for combining probabilistic cytoarchitectonic maps and functional imaging data. Neuroimage 25: 1325-1335. doi:10.1016/j.neuroimage.2004.12.034. PubMed: 15850749. 15850749

[51] Amunts K, Kedo O, Kindler M, Pieperhoff P, Mohlberg H et al. (2005) Cytoarchitectonic mapping of the human amygdala, hippocampal region and entorhinal cortex: intersubject variability and probability maps. Anat Embryol 210: 343-352. doi:10.1007/s00429-005-0025-5. PubMed: 16208455. 16208455

[52] Killgore WD, Yurgelun-Todd DA (2001) Sex differences in amygdala activation during the perception of facial affect. Neuroreport 12: 2543-2547. doi:10.1097/00001756-200108080-00050. PubMed: 11496145. 11496145

[53] Perach-Barzilay N, Tauber A, Klein E, Chistyakov A, Ne'eman R et al. (2013) Asymmetry in the dorsolateral prefrontal cortex and aggressive behavior: A continuous theta-burst magnetic stimulation study. Soc Neuroscience 8: 178-188. doi:10.1080/17470919.2012.720602. PubMed: 22963204. 22963204

[54] Gottesman II, Gould TD (2003) The endophenotype concept in psychiatry: Etymology and strategic intentions. Am J Psychiatry 160: 636-645. doi:10.1176/appi.ajp.160.4.636. PubMed: 12668349. 12668349

[55] Asahi S, Okamoto Y, Okada G, Yamawaki S, Yokota N (2004) Negative correlation between right prefrontal activity during response inhibition and impulsiveness: A fMRI study. Eur Arch Psy Clin N 254: 245-251. PubMed: 15309395. 10.1007/s00406-004-0488-z 15309395

[56] Gansler DA, Lee AK, Emerton BC, D'Amato C, Bhadelia R et al. (2011) Prefrontal regional correlates of self-control in male psychiatric patients: Impulsivity facets and aggression. Psychiatry Res Neuroimaging 191: 16-23. doi:10.1016/j.pscychresns.2010.09.003. PubMed: 21145213. 21145213

[57] Carter CS, Botvinick MM, Cohen JD (1999) The contribution of the anterior cingulate cortex to executive processes in cognition. Rev Neurosci 10: 49-67. PubMed: 10356991. 10356991 10.1515/revneuro.1999.10.1.49

[58] Kerns JG, Cohen JD, MacDonald AW, Cho RY, Stenger VA et al. (2004) Anterior cingulate conflict monitoring and adjustments in control. Science 303: 1023-1026. doi:10.1126/science.1089910. PubMed: 14963333. 14963333

[59] Sanfey AG, Rilling JK, Aronson JA, Nystrom LE, Cohen JD (2003) The neural basis of economic decision-making in the ultimatum game. Science 300: 1755-1758. doi:10.1126/science.1082976. PubMed: 12805551. 12805551

[60] Li C-s, Kosten TR, Sinha R (2006) Antisocial personality and stress-induced brain activation in cocaine-dependent patients. Neuroreport 17: 243-247. doi:10.1097/01.wnr.0000199471.06487.a2. PubMed: 16462591. 16462591

[61] Blair RJR, Morris JS, Frith CD, Perrett DI, Dolan RJ (1999) Dissociable neural responses to facial expressions of sadness and anger. Brain 122: 883-893. doi:10.1093/brain/122.5.883. PubMed: 10355673. 10355673

[62] Herpertz SC, Werth U, Lukas G, Qunaibi M, Schuerkens A et al. (2001) Emotion in criminal offenders with psychopathy and borderline personality disorder. Arch Gen Psychiatry 58: 737–745. doi:10.1001/archpsyc.58.8.737. PubMed: 11483139. 11483139

[63] Kimbrell TA, George MS, Parekh PI, Ketter TA, Podell DM et al. (1999) Regional brain activity during transient self-induced anxiety and anger in healthy adults. Biol Psychiatry 46: 454-465. doi:10.1016/S0006-3223(99)00103-1. PubMed: 10459394. 10459394

[64] Hornak J, Bramham J, Rolls ET, Morris RG, O'Doherty J et al. (2003) Changes in emotion after circumscribed surgical lesions of the orbitofrontal and cingulate cortices. Brain 126: 1691-1712. doi:10.1093/brain/awg168. PubMed: 12805109. 12805109

[65] Hornak J, Rolls ET, Wade D (1996) Face and voice expression identification inpatients with emotional and behavioural changes following ventral frontal lobe damage. Neuropsychologia 34: 247-261. doi:10.1016/0028-3932(95)00106-9. PubMed: 8657356. 8657356

[66] Tranel D, Bechara A, Denburg NL (2002) Asymmetric functional roles of right and left ventromedial prefrontal cortices in social conduct, decision-making, and emotional processing. Cortex 38: 589-612. doi:10.1016/S0010-9452(08)70024-8. PubMed: 12465670. 12465670

[67] Blair RJR (2007) The amygdala and ventromedial prefrontal cortex in morality and psychopathy. Trends Cogn Sci 11: 387-392. doi:10.1016/j.tics.2007.07.003. PubMed: 17707682. 17707682

[68] Blair RJR (2008) The amygdala and ventromedial prefrontal cortex: Functional contributions and dysfunction in psychopathy. Philos T Roy Soc 363: 2557-2565. doi:10.1098/rstb.2008.0027. PubMed: 18434283. PMC2606709 18434283

[69] Gordon HL, Baird AA, End A (2004) Functional differences among those high and low on a trait measure of psychopathy. Biol Psychiatry 56: 516-521. doi:10.1016/j.biopsych.2004.06.030. PubMed: 15450788. 15450788

[70] Berkowitz RL, Coplan JD, Reddy DP, Gorman JM (2007) The human dimension: How the prefrontal cortex modulates the subcortical fear response. Rev Neurosci 18: 191-207. PubMed: 18019606. 18019606 10.1515/revneuro.2007.18.3-4.191

[71] Hariri AR, Mattay VS, Tessitore A, Fera F, Weinberger DR (2003) Neocortical modulation of the amygdala response to fearful stimuli. Biol Psychiatry 53: 494-501. doi:10.1016/S0006-3223(02)01786-9. PubMed: 12644354. 12644354

[72] Silbersweig D, Clarkin JF, Goldstein M, Kernberg OF, Tuescher O et al. (2007) Failure of Frontolimbic Inhibitory Function in the Context of Negative Emotion in Borderline Personality Disorder. Am J Psychiatry 164: 1832–1841. doi:10.1176/appi.ajp.2007.06010126. PubMed: 18056238. 18056238

[73] Kiehl KA, Smith AM, Hare RD, Mendrek A, Forster BB et al. (2001) Limbic abnormalities in affective processing by criminal psychopaths as revealed by functional magnetic resonance imaging. Biol Psychiatry 50: 677-684. doi:10.1016/S0006-3223(01)01222-7. PubMed: 11704074. 11704074

[74] Osumi T, Nakao T, Kasuya Y, Shinoda J, Yamada J et al. (2012) Amygdala dysfunction attenuates frustration-induced aggression in psychopathic individuals in a non-criminal population. J Affect Disord 142: 331-338. doi:10.1016/j.jad.2012.05.012. PubMed: 22840629. 22840629

[75] Preacher KJ, Rucker DD, MacCallum RC, Nicewander WA (2005) Use of the extreme groups approach: A critical reexamination and new recommendations. Psychol Methods 10: 178-192. doi:10.1037/1082-989X.10.2.178. PubMed: 15998176. 15998176

[76] Gelman A, Park DK (2009) Splitting a predictor at the upper quarter or third and the lower quarter or third. Am Stat 63: 1-8. doi:10.1198/tast.2009.0001.

[77] Lobbestael J, Arntz A, Wiers RW (2008) How to push someone’s buttons: A comparison of four anger-induction methods. Cogn Emotion 22: 353-373. doi:10.1080/02699930701438285.

[78] Stemmler G (2010) Somatovisceral activation during anger. In: Potegal M International handbook of anger. New York: Springer Verlag pp. 103-121.

[79] Lehrl S, editor (1995) Mehrfachwahl-Wortschatz-Intelligenztest: MWT-B [Multiple Choice Vocabulary Test]. Balingen: Perimed-spitta.

[80] Reitan RM, Wolfson D, editors (1985) The Halstead–Reitan Neuropsychological Test Battery: Therapy and clinical interpretation. AZ.: Tucson.

[81] Aschenbrenner A, Tucha O, Lange K, editors (2000) RWT Regensburger Wortflüssigkeits-Test. Handanweisung. Goettingen: Hogrefe.

