# Supplementary material for: Anger under Control: Neural Correlates of Frustration as a Function of Trait Aggression
Source: PLoS One. 2013 Oct 18;8(10):e78503. doi: 10.1371/journal.pone.0078503 (PMC3799631; doi:10.1371/journal.pone.0078503)
Supplement: Table S1 — Activations for the main effect of condition, p <.001 uncorr., k=20. (DOCX) [file pone.0078503.s001.docx]

|  | **MNI Coordinates** | | |  |  |  |  |
| --- | --- | --- | --- | --- | --- | --- | --- |
| **Brain region** | **x** | **y** | **z** | **side** | **k** | **Z-score** | **p-value** |
| anterior cingulate cortex | 2 | 30 | 10 | R | 9545 |  | 0.000 |
|  | 0 | 12 | -6 |  | 81 | 4.18 | 0.000 |
| middle cingulate gyrus | 4  4 | -4  -6 | 36  0 | R | 274 | 4.62  4.07 | 0.000 |
| posterior cingulate cortex | 16 | -34  -6 | 28  0 | R | 23 | 3.51 | 0.000 |
| superior frontal cortex | -2 | 2 | 64 | L | 977 | 4.64  3.80 | 0.000 |
|  | 10 | 32 | 64 | R | 30 | 3.65 | 0.000 |
| middle frontal cortex | -22 | 16 | 34 | L | 31 | 4.37 | 0.000 |
| precentral gyrus  eft lateral globus pallidus | -54 | -2 | 48 | L | 268 | 4.21 | 0.000 |
|  | -62 | 0 | 30 | L | 37 | 3.87 | 0.000 |
|  | 36 | -24 | 56 | R | 208 | 3.84 | 0.000 |
| angular gyrus | -46 | -70 | 38 | L | 2139 | 6.58 | 0.000 |
|  | 50 | -74 | 34 | R | 1049 | 5.99 | 0.000 |
| superior parietal cortex | -26 | -56 | 46 | L | 727 | 4.48 | 0.000 |
|  | 32 | -44 | 46 | R | 39 | 3.77 | 0.000 |
| precuneus | 22 | -68 | 40 | R | 417 | 4.02 | 0.000 |
| parahippocampal gyrus | -28 | -34 | -14 | L | 5886 | 6.30  3.50 | 0.000 |
| hippocampus | 26 | -6 | -20 | R | 3370 | 5.93 | 0.000 |
| cerebellum  left claustrum | 28 | -84 | -34 | R | 451 | 4.33  4.07 | 0.000 |
|  | 6 | -50 | -46 | R | 170 | 4.17 | 0.000 |
|  | -26 | -82 | -30 | L | 28 | 3.56 | 0.000 |
| striatum | 0 | -10 | 8 |  | 23 | 3.35 | 0.000 |

Abbreviations: k = cluster size
