# Supplementary material for: Anger under Control: Neural Correlates of Frustration as a Function of Trait Aggression
Source: PLoS One. 2013 Oct 18;8(10):e78503. doi: 10.1371/journal.pone.0078503 (PMC3799631; doi:10.1371/journal.pone.0078503)
Supplement: Table S2 — Activations for the main effect of group, p <.001 uncorr., k=20. (DOCX) [file pone.0078503.s002.docx]

|  | **MNI coordinates** | | |  |  |  |  |
| --- | --- | --- | --- | --- | --- | --- | --- |
| **Brain Region** | **x** | **y** | **z** | **side** | **k** | **Z-score** | **p-value** |
| vlPFC/ dlPFC | -52 | 26 | 32 | L | 189 |  | 0.000 |
| dlPFC | 44 | 52 | 18 | R | 28 | 3.60 | 0.000 |
|  | 48 | 20 | 28 | R | 32 | 3.40 | 0.000 |
| precentral gyrus | -40 | 2 | 58 | L | 30 | 3.66 | 0.000 |
| supplementary motor area (SMA) | -6 | 22 | 44 | L | 38 | 3.59  3.80 | 0.000 |
| middle cingulate cortex  eft lateral globus pallidus | 10 | 30 | 30 | R | 55 | 3.58 | 0.000 |
| insula | 24 | 24 | 4 | R | 133 | 3.94 | 0.000 |
|  | 38 | 22 | -4 | R | 37 | 3.51 | 0.000 |
| middle temporal cortex  left claustrum | -54 | -42 | 4 | L | 61 | 3.73  4.07 | 0.000 |
| caudate | -16 | 16 | 8 | L | 136 | 3.16 | 0.000 |
|  | -20 | 0 | 18 | L | 39 | 3.43 | 0.000 |
| globus pallidus | 12 | 4 | 0 | R | 36 | 3.40  3.80 | 0.000 |
| cerebellum | 16 | -82 | -18 | R | 22 | 3.45 | 0.000 |
| fusiform gyrus | -28 | -68 | -12 | L | 89 | 3.73 | 0.000 |
| occipital lobe | 20 | -92 | 30 | R | 58 | 3.84 | 0.000 |
|  | 14 | -88 | 12 | R | 60 | 3.59 | 0.000 |
|  | 34 | -86 | -8 | R | 36 | 3.33 | 0.000 |
|  | -24 | -92 | 6 | L | 74 | 3.65 | 0.000 |

Abbreviations: k = cluster size
