# Supplementary figures and images for: Anger under Control: Neural Correlates of Frustration as a Function of Trait Aggression
Source: PLoS One. 2013 Oct 18;8(10):e78503. doi: 10.1371/journal.pone.0078503 (PMC3799631; doi:10.1371/journal.pone.0078503)

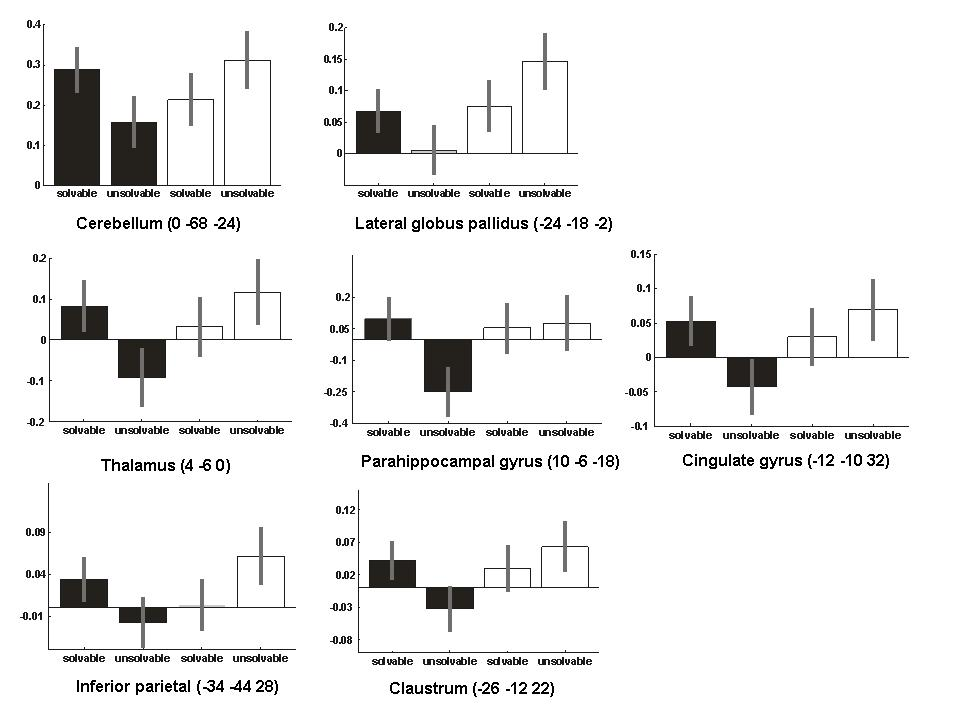

Supplement: Figure S1 — Parameter estimate plots for the activation cluster of the group X condition interaction. (DOCX) [file pone.0078503.s003.docx]
